# Changes and determinants of health-related quality of life among people newly diagnosed with HIV in China: a 1-year follow-up study

**DOI:** 10.1007/s11136-018-1998-x

**Published:** 2018-09-11

**Authors:** Yunxiang Huang, Dan Luo, Xi Chen, Dexing Zhang, Min Wang, Yangyang Qiu, Ying Liu, Bihua Peng, Lu Niu, Shuiyuan Xiao

**Affiliations:** 1 Department of Social Medicine and Health Management, Xiangya School of Public Health, Central South University, Changsha, Hunan People’s Republic of China; 2 Hunan Provincial Center for Disease Prevention and Control, Changsha, Hunan People’s Republic of China; 3 The Jockey Club School of Public Health and Primary Care, Faculty of Medicine, The Chinese University of Hong Kong, Shatin, Hong Kong People’s Republic of China; 4 HIV/AIDS Research Institute, The First Hospital of Changsha, Changsha, Hunan People’s Republic of China

**Keywords:** Health-related quality of life, People newly diagnosed with HIV, Changes, Determinants

## Abstract

**Purpose:**

This study aimed to investigate changes in health-related quality of life (HRQoL) among people newly diagnosed with HIV and to identify factors associated with HRQoL.

**Methods:**

Newly diagnosed HIV-positive individuals were consecutively recruited and assessed at baseline and 1-year follow-up after diagnosis. HRQoL was measured through the physical health summary score (PHS) and mental health summary score (MHS) derived from the Medical Outcomes Study HIV Health Survey. Socio-demographic, clinical, and psychological information was also collected at both times. Generalized estimating equations were applied to explore factors associated with HRQoL in 1 year.

**Results:**

A total of 410 participants were included. After 1 year, significant increases were observed for both the mean PHS score (53.5–55.0; *p* = 0.009) and the mean MHS score (44.2–49.0; *p* < 0.001). Older age (*p* = 0.024), rural household registration (*p* = 0.031), HIV-related symptoms (*p* < 0.001), and depression (*p* = 0.014) were negatively associated with PHS. Additionally, the negative association between stress and PHS increased over time (*β* = − 0.07 for the baseline; *β* = − 0.18 for the 12-month follow-up; *p* < 0.001). HIV-related symptoms, depression, lower social support, and higher levels of stress (all *p* < 0.001) were negatively associated with MHS. Additionally, the negative relationship between stress and MHS was stronger among participants who were asymptomatic (*p* = 0.015).

**Conclusion:**

A relatively lower HRQoL among HIV-infected people shortly after HIV diagnosis and an increase in HRQoL among people 1 year after HIV diagnosis were observed. Additional attention should be paid to individuals of older age, from rural areas, with HIV-related symptoms, with depression, with high levels of stress, and with a lack of social support.

## Introduction

It has been estimated that by the end of 2017, a cumulative total of 758,610 people were living with HIV in China, with increasing HIV cases over the years [1]. As the Chinese government scaled up the National Free Antiretroviral Treatment Programme (NFATP) in 2003, approximately 542,000 people in China were receiving antiretroviral therapy (ART) by the end of June 2017 [2]. A dramatic decline in mortality and morbidity has been found among HIV-infected individuals [3]. HIV/AIDS infection is more often a chronic manageable disease rather than a terminal illness [4]. Consequently, the focus of HIV–AIDS care has been shifting from increasing life expectancy to the improving health-related quality of life (HRQoL) [5].

HRQoL is a multidimensional concept that includes factors such as physical health, psychological health, social functioning, and perception about general health [6]. In addition, HRQoL is a subject concept that could vary at different time points [7]. Regularly assessing HRQoL and identifying determinants of HRQoL among HIV-positive individuals can help us to discover potential problems that influence health and can provide important information to help healthcare providers and policy makers improve HRQoL.

Many previous studies have investigated factors related to HRQoL among HIV-positive individuals. An existing literature review reported that determinants of HRQoL among HIV-infected individuals could be divided into four categories: socio-demographic, clinical, psychosocial, and behavior factors [7]. Socio-demographic factors such as gender, age, education, and employment were important predictors of HRQoL. Clinical factors such as the presence of symptoms, lower levels of CD4 cell counts, and an advanced stage of disease were also associated with worse HRQoL. Moreover, psychosocial problems including depression, anxiety, and high stress can negatively affect HRQoL, but active coping styles and social support are regarded as a mediator to buffer the negative influence of psychological distress on HRQoL. Correlations between HRQoL and behavior factors such as adherence to ART, alcohol and drug use, smoking, and risky sexual behaviors were also demonstrated.

There have already been many studies on the determinants of HRQoL in HIV-infected individuals in China, while most have used a cross-sectional, rather than longitudinal study design [8–10]. Although longitudinal studies have been conducted in other countries (e.g., Uganda, the US), they have focused primarily on HIV patients receiving ART, who tend to be at a more advanced stage of the disease [11–13]. However, this population does not represent the overall population living with HIV. In addition, determinants of HRQoL may differ depending on the disease stage. Unfortunately, to the best of our knowledge, few studies have examined changes and determinants of HRQoL in the newly diagnosed HIV-positive population.

Being diagnosed with HIV is a stressful life event for most people [14, 15]. People newly diagnosed with HIV usually experience many stressors such as physical health, psychosocial adjustment, stigma, and side effects of treatment [15]. They also struggle with psychosocial problems such as depression, anxiety, and a lack of social support [16, 17]. All these factors may negatively influence HRQoL [18, 19]. O’Keefe and Wood found that the majority of decreases in HRQoL, particular in the psychological domain, occurred in early stages of HIV progression [20]. Therefore, assessing changes in HRQoL and identifying factors that influence HRQoL among people newly diagnosed with HIV should not be ignored.

In summary, there exists a significant knowledge gap regarding the changes and determinants of HRQoL among people newly diagnosed with HIV, especially those from China. Therefore, this study aims to investigate changes in HRQoL among people newly diagnosed with HIV and to identify factors that influence HRQoL.

## Methods

### Study design and population

This was a 1-year follow-up study. The baseline survey was conducted between March 1, 2013 and September 30, 2014. Then, a follow-up survey was completed 1 year after the baseline survey. We recruited people newly diagnosed with HIV (an enzyme-linked immunosorbent screening assay and a confirmatory Western Blot were conducted) from the Changsha Center for Disease Control and Prevention (CDC), Hunan Province, China. Participants were consecutively recruited if they (a) were aged 18 years or older; (b) received the diagnosis of HIV within 1 month before enrolling in this study (newly diagnosed with HIV); (c) had been living in Changsha for more than 6 months; and (d) communicated without cognitive impairment. Cognitive impairment was recognized by CDC staff using the Mini-mental State Examination (MMSE). Patients with suspected cognitive impairment were referred by relative CDC staff to a psychiatric hospital, for further confirmatory tests and treatment.

A self-administered questionnaire including HRQoL, social-demographic, clinical, and psychosocial characteristics was completed by eligible participants at baseline and 1-year follow-up. This study was approved by the Human Research Ethics Committee of Central South University, and all eligible participants provided written informed consent before participation.

### Measures

#### Socio-demographic characteristics

The socio-demographic characteristics included age (18–29, 30–39, or ≥ 40), gender, household registration (rural or urban), marital status (single, married, or divorced/widowed), education level (high school or lower, college or higher), employment (employed or unemployed), individual monthly income (‘≤ 4000 Yuan’ or ‘> 4000 Yuan,’ $626), and the mode of HIV transmission (heterosexual, homosexual, or others). The group of “others” included intravenous drug use (IDU), blood products, and uncertain means.

#### Clinical characteristics

We also surveyed clinical characteristics, including HIV-related symptoms, CD4 count, and ART status. Participants were asked in questionnaires whether they had the following HIV-related symptoms: persistent fever, diarrhea, expectoration or cough of an unknown origin for more than 1 month, unintentional weight loss of more than 10% in the last 3 months, active tuberculosis in the past 6 months, symptoms of oral thrush or recurrent herpes simplex, or other HIV-related symptoms. We obtained each participant’s CD4 count and ART status (whether they had received ART during follow-up period) from the Chinese HIV/AIDS Comprehensive Response Information Management System. The CD4 count was categorized into ≤ 200 cells/mm^3^, 200–500 cells/mm^3^, and > 500 cells/mm^3^. ART initiation during the follow-up period was documented at the 1-year follow-up survey.

#### Psychosocial characteristics

##### Depressive symptoms

Depressive symptoms were measured by the 9-item Patient Health Questionnaire (PHQ-9) [21]. Participants were asked how often they had suffered from 9 depressive symptoms during the last 2 weeks, using a 4-point Likert scale from 0 (not at all) to 3 (nearly every day). The total score ranges from 0 to 27, with a score ≥ 10 indicating the probable major depression disorder. A higher score indicates more severe depressive symptoms. The Chinese version of the PHQ-9 has a good validity and reliability with a Cronbach’s *α* coefficient of 0.86 [22].

##### Anxiety symptoms

Anxiety symptoms were based on the 7-item Generalized Anxiety Disorder questionnaire (GAD-7). The GAD-7 comprises a 4-point Likert scale from 0 (not at all) to 3 (nearly every day). The total score ranges from 0 to 21. Alongside the PHQ-9, a score of 10 or more on the GAD-7 was used as the cut-off for identifying generalized anxiety disorder [23]. A higher score indicates more severe anxiety symptoms. The Chinese version of the GAD-7 has a good validity and reliability, with a Cronbach’s *α* coefficient of 0.88 [24].

#### HIV-related stress

HIV-related stress was assessed using the HIV/AIDS stress scale (SS-HIV), a measure of stress specific to HIV/AIDS [25]. Participants were asked how much they had suffered from HIV/AIDS stress in the past month. The scale includes three dimensions: social stress, instrumental stress, and emotional/existential stress. Higher scores indicate higher levels of HIV-related stress. Our research group translated this scale into Chinese and validated it with the consent of the original author. The Chinese version of the HIV/AIDS stress scale (CSS-HIV) has a good validity and reliability, with an overall Cronbach’s *α* coefficient of 0.906 [26].

#### Social support

Social support was measured by the Social Support Rating Scale (SSRS), which is commonly used in China [27–29]. The scale contains three dimensions of social support: objective support, subjective support, and support usage [30]. The total score is calculated on the basis of these three dimensions, and ranges from 11 to 66 points. A higher score indicates a higher level of social support. In this study, the Cronbach’s *α* coefficient for the entire scale was 0.805.

#### Health-related quality of life

The HRQoL was measured using the Medical Outcomes Study HIV Survey (MOS-HIV) [31]. The MOS-HIV is a brief and comprehensive measurement of HRQoL and is widely used for people with HIV infection. The MOS-HIV Health survey contains 35 items and includes 11 dimensions, including general health perception, physical functioning, role functioning, pain, social functioning, mental health, energy, health distress, cognitive functioning, overall quality of life, and health transition. The scores of each dimension, which are obtained with standard scoring procedures, range from 0 to 100, with higher scores indicating a better quality of life. Moreover, the score can be summarized into two summary scores, a physical health summary score (PHS) and a mental health summary score (MHS). The simplified Chinese version of the MOS-HIV has reported good validity and reliability [32].

### Statistical analysis

All data were analyzed using SPSS22.0 (SPSS; Inc, Chicago, IL, USA). First, descriptive statistics were performed and presented as percentages, medians with interquartile ranges (IQR), means and standard deviations (SD). Second, we compared the baseline sample characteristics between the participants who completed both surveys and participants who dropped out of the follow-up survey, using Mann–Whitney tests for continuous variables and Chi-square tests for categorical variables. Third, paired *t* tests, Mann–Whitney tests and Chi-square tests were used to assess changes in HRQoL and time-dependent variables (including depression, anxiety, stress, social support, CD4 count, and HIV-related symptoms).

Finally, generalized estimating equations (GEE) were applied to identify the factors associated with PHS and MHS. Variables that were statistically associated with PHS or MHS with a *p* value ≤ 0.2 in the univariate linear regression with GEE were selected in the multivariate analysis [33]. Before the multivariate analyses, collinearity diagnosis was verified using eigenvalue and variance proportions. Consequently, to avoid multi-collinearity, anxiety was excluded from our final models. To further test for collinearity, we removed depression from the models, and we observed no changes in the *p* value for each of the other variables between the two models (with and without depression). Additionally, the interaction of time-dependent covariates with time (baseline versus 12 months) and the interaction between each statistically significant variable from the multivariable analysis were investigated in the GEE models. Only significant interactions were entered into the final GEE models. For all analyses, two-tailed *p* values < 0.05 were considered to be statistically significant.

## Results

### Characteristics of the study participants

A total of 855 people newly diagnosed with HIV were eligible for inclusion in this study. The final number of participants enrolled in the study was 557 (65.1%) at baseline. After 1 year, 2 (1.5%) participants had died, and 145 participants were withdrawn from the study for the following reasons: a geographic move (*n* = 35, 23.8%), a loss of contact (*n* = 35, 23.8%), and refusal to participate in the follow-up survey (*n* = 75, 51.0%). The final data used for this analysis were collected from 410 participants who had completed surveys at baseline and at the 1-year follow-up.

Of the 410 participants remaining in this study, 218 (53.2%) initiated ART during follow-up with a median total treatment duration of 6 months (IQR 4–9), and 48.8% had a rural household registration. Participants were predominantly male (91.7%) and single (61.5%), and had median age of 28 years (IQR 24–36). Over half of participants (56.1%) were infected with HIV through homosexual contact. The sample characteristics of the participants are shown in Table 1. Compared to the 410 participants who completed a 1-year follow-up survey, the 147 participants who did not complete a follow-up survey showed no significant difference in any baseline characteristic, except for showing a higher rate of employment (Table 1).

**Table 1:** Baseline sample characteristics of final participants (*n* = 410) and excluded participants (*n* = 147)

Characteristics	Completed follow-up survey *n* = 410 (%)	Lost to follow-up *n* = 147 (%)	*p* value
Sex			
Male	376 (91.7%)	139 (94.6%)	0.261^a^
Female	34 (8.3%)	8 (5.4%)	
Age, median (IQR)	28 (24–36)	29 (24–38)	0.538^b^
18–29	228 (55.6%)	87 (59.2%)	
30–39	96 (23.4%)	28 (19.0%)	
≥ 40	86 (21.0%)	32 (21.8%)	
Marital status			
Married	110 (26.8%)	29 (19.7%)	0.159^a^
Divorced/widowed	48 (11.7%)	23 (15.6%)	
Single	252 (61.5%)	95 (64.6%)	
Residence			
Urban	210 (50.2%)	73 (47.9%)	0.746^a^
Rural	200 (48.8%)	74 (50.3%)	
Education			
Senior or lower	222 (51.1%)	80 (54.4%)	0.954^a^
College or higher	188 (45.9%)	67 (45.6%)	
Employment			
Yes	276 (67.3%)	115 (78.2%)	0.013^a^
No	134 (32.7%)	32 (21.8%)	
Income (RMB)			
≤ 4000	259 (63.2%)	80 (54.4%)	0.062^a^
> 4000	151 (36.8%)	67 (45.6%)	
HIV transmission			
Heterosexual	172 (42.0%)	54 (36.7%)	0.507^a^
Homosexual	230 (56.1%)	92 (62.6%)	
Other	8 (1.9%)	1 (0.7%)	
CD4 count (cells/mm^3^), median (IQR)	357 (254–471)	350 (258–458)	0.930^b^
< 200	62 (15.1%)	15 (10.2%)	
200–500	264 (64.4%)	105 (71.4%)	
> 500	84 (20.5%)	27 (18.4%)	
HIV-related symptoms			
Yes	153 (37.3%)	46 (31.3%)	0.191^a^
No	257 (62.7%)	101 (68.7%)	
PHQ-9 scores (0–27)			
No depression (< 10)	249 (60.7%)	97 (66.0%)	0.260^a^
Probable depression (≥ 10)	161 (39.3%)	50 (34.0%)	
GAD-7 scores (0–21)			
No anxiety (< 10)	286 (69.8%)	104 (70.7%)	0.822^a^
Probable anxiety (≥ 10)	124 (30.2%)	43 (29.3%)	
HIV stress, median (IQR)	26 (15–40)	25 (14–38)	0.339^b^
Social support, median (IQR)	29 (24–24)	30 (22–34)	0.852^b^

^a^Chi-square test

^b^Mann–Whitney test

### Changes in health quality of life

A significant improvement was observed for both PHS and MHS in HIV-infected individuals 1 year after being diagnosed with HIV (Table 2). PHS improved by 1.1, from 53.9 (SD = 7.1) at baseline to 55.0 (SD = 6.5) at follow-up (*p* < 0.01). Additionally, the mean MHS scores increased by 4.8, with a mean score of 44.2 (SD = 10.1) at baseline to 49.0 (SD = 9.3) at follow-up (*p* < 0.001). After 1 year, there was an increase in the mean score of each domain compared to the mean score at baseline. The largest increases were in health transition (13.3 points) and health distress (12.4 points). The smallest increases occurred in the physical function (1.2 points) and role function (2.6 points). All dimensions of the HRQoL increased significantly after 1 year, except for physical and role function. Table 2 shows the details of the changes in HRQoL.

**Table 2:** Changes in MOS-HIV subscales and summary scores between baseline and follow-up

HRQoL	Baseline		Follow-up		Difference		*p* value^a^
	Mean	SD	Mean	SD	Mean	SD	
Summary score							
PHS	53.9	7.1	55.0	6.5	1.0	7.8	0.009
MHS	44.2	10.1	49.0	9.3	4.9	11.2	< 0.001
Dimensions							
General health perception	51.5	19.3	57.9	19.1	6.4	21.6	< 0.001
Physical function	90.7	15.1	91.8	13.9	1.2	18.7	0.210
Role function	89.3	27.7	90.9	27.0	2.6	33.4	0.076
Social function	75.9	29.0	82.0	24.9	6.1	34.8	< 0.001
Cognitive function	75.0	21.6	79.4	20.2	4.4	26.1	0.001
Pain	83.6	16.9	87.0	15.3	3.4	20.3	0.001
Mental health	61.9	19.3	70.2	21.3	8.4	25.3	< 0.001
Energy/fatigue	59.8	20.4	63.8	18.1	4.0	22.0	< 0.001
Health distress	64.7	25.3	77.0	22.4	12.4	28.9	< 0.001
Quality of life	55.0	20.1	64.2	15.7	9.2	22.8	< 0.001
Health transition	42.4	24.9	55.7	23.9	13.3	31.7	< 0.001

^a^The *p* value denotes the significance of the difference between the baseline and 1-year follow-up using paired *t* tests

### Changes in psychosocial and clinical characteristics

After 1 year of being diagnosed with HIV, the prevalence of probable depression and anxiety decreased from 39.3 to 30.2% at baseline to a rate of 16.1% and 12.2% at follow-up (both *p* < 0.001), respectively. A significant decrease in the median score was observed in both HIV-related stress and social support (*p* < 0.001 for stress, *p* = 0.017 for social support). The median baseline CD4 count was 357 cells/mm^3^ (IQR 254–471), and 416 cells/mm^3^ (IQR 303–517) at 1-year follow-up (*p* < 0.001). The presence of symptoms decreased from 37.3% at baseline to 27.1% at follow-up (*p* = 0.002). Table 3 shows the details of the changes in psychosocial and clinical factors.

**Table 3:** Changes in psychosocial and clinical characteristics between baseline and follow-up

Time-dependent variables	Baseline (*n* = 410)	Follow-up (*n* = 410)	*p* value
Clinical variables			
CD4 cell count, median (IQR)	357 (254–471)	416 (303–517)	< 0.001^a^
HIV-related symptoms, *n* (%)			
Yes	153 (37.3%)	111 (27.1%)	0.002^b^
No	257 (62.7%)	299 (72.9%)	
Psychosocial variables			
Probable depression, *n* (%)			
Yes	161 (39.3%)	66 (16.1%)	< 0.001^b^
No	249 (60.7%)	344 (83.9%)	
Probable anxiety, *n* (%)			
Yes	124 (30.2%)	50 (12.2%)	< 0.001^b^
No	286 (69.8%)	360 (87.8%)	
HIV-related stress	26.5 (16–40)	15 (8–26)	< 0.001^a^
Social support	29 (24–34)	27 (22–34)	0.012^a^

^a^Mann–Whitney tests

^b^Chi-square test

### Factors associated with health-related quality of life

All variables were included in the final multivariate models because all variables were found to be correlated with PHS or MHS in a univariate linear regression with GEE at *p* ≤ 0.20 (Table 4).

Factors significantly associated with the PHS score in the multivariate GEE analysis were age, household registration, mode of HIV transmission, HIV-related symptoms, depression, and HIV-related stress. Participants aged 40 years and older had a lower PHS score of 1.84 points on average compared to those below 30 years of age (*p* = 0.024). Participants with a rural household registration scored 1.04 points lower on average than urban household (*p* = 0.031). Participants who were infected by IDU, blood products, or by uncertain means had a PHS score of 5.64 points, lower than that for participants infected by heterosexual transmission (*p* = 0.004). Participants who experienced HIV-related symptoms scored 2.54 points lower on average than those who reported no HIV-related symptoms (*p* < 0.001). Participants with probable depression scored 1.59 points lower on average than those with no depression (*p* = 0.014). A higher level of stress was associated with lower PHS score and with a greater reduction in PHS over time (*β* = − 0.07 for baseline; *β* = − 0.18 for 12-month follow-up; *p* < 0.001).

**Table 4:** Associations between participant characteristics and HRQoL using univariate linear regression with GEE

Characteristics	PHS		MHS	
	*β* coefficient (95% CI)	*p* value	*β* coefficient (95% CI)	*p* value
Study visit				
Baseline				
Follow-up	1.03 (0.27 to 1.78)	0.008	4.88 (3.80 to 5.97)	< 0.001
Sex				
Male	Ref		Ref	
Female	− 2.79 (− 5.92 to 0.35)	0.082	0.38 (− 2.50 to 0.36)	0.796
Age in years				
18–29	Ref		Ref	
30–39	− 0.20 (− 1.69 to 1.28)	0.789	− 0.39 (− 2.64 to 1.37)	0.781
≥ 40	− 1.97 (− 3.67 to − 0.27)	0.023	0.33 (− 1.98 to 2.63)	0.737
Household registration				
Urban	Ref		Ref	
Rural	− 1.03 (− 2.28 to 2.22)	0.106	− 0.29 (− 2.09 to 1.50)	0.749
Marital status				
Married	Ref		Ref	
Single/separated	0.94 (− 0.39 to 2.26)	0.162	0.12 (− 1.94 to 2.19)	0.907
Divorced/widowed	− 0.78 (− 3.18 to 1.61)	0.522	− 1.14 (− 4.39 to 2.11)	0.493
Education				
Senior or lower	Ref		Ref	
College or higher	− 1.18 (− 2.41 to 0.05)	0.06	− 0.76 (− 2.53 to 1.00)	0.396
Employment				
Yes	Ref		Ref	
No	− 1.40 (− 2.84 to 0.04)	0.056	− 0.19 (− 2.13 to 1.74)	0.845
Monthly income (RMB)				
≤ 4000	Ref		Ref	
> 4000	2.31 (1.12 to 3.51)	< 0.001	1.24 (− 0.55 to 3.02)	0.175
HIV transmission				
Heterosexual	Ref		Ref	
Homosexual	0.49 (0.83 to 1.80)	0. 470	− 0.25 (− 2.09 to 1.59)	0.792
Other	− 1.97 (− 4.20 to 0.26)	0.083	− 1.69 (− 8.42 to 5.05)	0.623
CD4 count (cells/mm^3^)				
< 200	Ref		Ref	
200–500	2.07 (− 0.62 to 4.76)	0.132	1.37 (− 1.68 to 4.43)	0.378
> 500	3.00 (0.22 to 5.79)	0.035	2.18 (1.25 to 5.61)	0.212
HIV symptom				
Yes	Ref		Ref	
No	4.50 (3.07 to 5.92)	< 0.001	5.30 (3.33 to 7.28)	< 0.001
ART				
Yes	Ref		Ref	
No	− 0.33 (0.51 to − 1.32)	0.516	− 4.23 (− 5.65 to − 2.82)	< 0.001
Depressive symptoms				
No depression (< 10)	Ref		Ref	
Probable depression (≥ 10)	− 7.50 (− 9.53 to − 5.47)	< 0.001	− 11.71 (− 13.90 to − 5.93)	< 0.001
HIV-related stress	− 0.22 (− 0.26 to − 0.17)	< 0.001	− 0.41 (− 0.46 to − 0.37)	< 0.001
Social support	0.11 (0.04 to 0.18)	< 0.001	0.30 (0.20 to 0.39)	< 0.001

MHS score was associated with HIV-related symptoms, depression, social support, and HIV-related stress in the multivariate GEE analysis. Participants who reported probable depression had a lower MHS score by 4.68 points on average (*p* < 0.001), and participants who presented no HIV-related symptoms scored 4.15 points higher on average than participants who experienced HIV-related symptoms (*p* < 0.001). An increase of one unit in social support was associated with a 0.12-point increase in the MHS score (*p* < 0.001). An increase of one unit in stress was associated with a 0.26-point decrease in the MHS score (*p* < 0.001). A significant interaction was found between HIV-related symptoms and stress. The negative relationship between stress and MHS was stronger for participants who were asymptomatic (*p* = 0.015). Details on the factors associated with HRQoL are shown in Table 5.

**Table 5:** Associations between participant characteristics and HRQoL using multivariate linear regression with GEE

Characteristics	PHS		MHS	
	*β* coefficient (95% CI)	*p* value	*β* coefficient (95% CI)	*p* value
Age				
18–29	Ref			
30–39	− 0.64 (− 1.85 to 0.56)	0.295		
≥ 40	− 2.07 (− 3.67 to − 0.47)	0.011		
Household registration				
Urban	Ref			
Rural	− 1.05 (− 1.99 to − 0.12)	0.027		
HIV-related symptoms				
Yes	Ref		Ref	
No	2.54 (1.60 to 3.47)	< 0.001	4.23 (2.03 to 6.42)	< 0.001
PHQ-9 scores (0–27)				
No depression (< 10)	Ref		Ref	
Probable depression (≥ 10)	− 1.76 (− 3.02 to − 0.50)	0.006	− 4.62 (− 5.92 to − 3.33)	< 0.001
HIV-related stress	− 0.07 (− 0.11 to − 0.02)	0.003	− 0.25 (− 0.31 to − 0.19)	< 0.001
Social support	0.03 (− 0.02 to 0.08)	0.258	0.13 (0.06 to 0.19)	< 0.001
Symptom × stress				
Yes			Ref	
No			− 0.08 (− 0.15 to − 0.02)	0.012
Time × stress				
Baseline	Ref			
Follow-up	− 0.11 (− 0.16 to − 0.06)	< 0.001		

## Discussion

In this study, we explored the changes in and determinants of HRQoL among HIV-positive individuals who were at an early stage of disease progression. We found a relatively lower HRQoL among HIV-infected people shortly after HIV diagnosis and a significant increase in both physical and mental aspects of HRQoL 1 year after HIV diagnosis. Older age, rural household registration, HIV-related symptoms, depression, and higher levels of HIV-related stress were negatively associated with PHS. Additionally, HIV-related symptoms, depression, higher levels of stress, and less social support were negatively associated with MHS.

The baseline PHS in our sample was relatively higher, and MHS was lower than in other studies with cross-sectional or longitudinal designs [34–38]. The baseline PHS in our study was higher than in other studies likely because the participants included in our study were newly diagnosed with HIV and had relatively good physical health. Most participants in our study were asymptomatic and had relatively higher levels of CD4 counts, with two-thirds of participants reporting an absence of symptoms and only 15.1% of participants having CD4 counts < 200 cell/mm^3^ at baseline. Additionally, the relatively higher levels of CD4 counts in most participants might explain why we did not observe an association between CD4 counts and HRQoL.

The relatively lower baseline score of MHS in our study might be due to the negative illness appraisals after the diagnosis of HIV infection. A qualitative study of illness appraisals in people newly diagnosed with HIV indicated that nearly half of participants interpreted HIV diagnosis to mean death or stigma [39]. Negative illness appraisals might lead these participants to perceive themselves to have poor mental health. Additionally, the news of HIV sero-positive status could be a great stressor to most individuals, and might negatively influence the mental health of people newly diagnosed with HIV [14, 16].

A significant increase in both physical and mental dimensions of HRQoL was observed among participants after 1 year of HIV diagnosis, similar to the longitudinal trend of HRQoL in the ART era reported by previous studies [40, 41]. Some investigators have proposed that individuals’ goals and concerns will change over time according to their disease stage [42]. At baseline, they might want to return to their health status before infection, and therefore, they might appraise HRQoL as low. At a later stage, their goal might turn to maintaining their current health condition, thereby changing their perceptions of HRQoL. Additionally, people will become accustomed to or accept the disease and learn how to cope with the negative impact of the disease as time passes after diagnosis [35]. The above results from our study emphasize that we should pay attention to longitudinal changes rather than cross-sectional assessments of HRQoL.

For this study sample, at baseline, nearly 39.3% of the participants scored ≥ 10 on the PHQ-9 scale (indicating a probable major depression disorder), which was higher than the percentage at the follow-up survey. The overwhelming news of HIV diagnosis might precipitate the risk of depression [14, 43]. In agreement with other studies, we found that depression had a negative impact on HRQoL [44–46]. A longitudinal study among men with HIV infection indicated that baseline depressive symptoms were significantly predictive of deterioration in HRQoL at 1-year follow-up [47]. In our study, we proved using both baseline and follow-up data that depression was associated with worsened HRQoL. These findings all demonstrated the importance of the early detection and treatment of depressive symptoms for improving HRQoL among people with HIV.

Consistent with cross-sectional studies in Nepal and Hong Kong, this study found that higher levels of stress were associated with worse HRQoL [18, 48]. The fact that HIV-positive individuals experienced higher levels of stress than the general population has been consistently documented by many previous studies [49]. Moreover, we found that the negative association between HIV-related stress and PHS increased over time. A chronic exposure to high levels of stress has been previously reported to decrease immunity, lead to increased symptomatology, and hasten disease progression to AIDS [50], which may contribute to the increased negative impact of stress on PHS at follow-up. These findings suggest that regularly assessing and managing stress across the disease trajectory should be included as an essential strategy in interventions.

A positive association between social support and HRQoL has been found in many previous studies [51, 52]. The results of this investigation also suggest that a higher level of social support was strongly related to better mental health. In our early report, we applied path analysis with structural equation modeling to demonstrate that social support may directly contribute to better HRQoL and that it had an indirect role in improving HRQoL by reducing depression and stress [53]. However, some HIV-positive individuals are more likely to isolate themselves from family, friends, or colleagues because of stigma, ultimately resulting in a lack of social support [54]. The potential positive influence of social support on HRQoL should not be ignored. Enhancing social support in this population should be considered as an important intervention to improve HRQoL.

We found a strong relationship between the presence of HIV-related symptoms and HRQoL, with asymptomatic participants having higher scores in PHS and MHS. Considerable studies had demonstrated that HIV-related symptoms had a detrimental impact on HRQoL [11, 55, 56]. A prospective study of HIV-infected patients in the US reported that changes in HRQoL were significantly and consistently influenced by changes in symptoms over 18 months [57]. Therefore, routine diagnosis and appropriate treatments of symptoms among HIV-positive individuals are key elements to improve HRQoL.

Our results on the interaction between stress and HIV-related symptoms on MHS may need further attention and exploration. Surprisingly, the negative relationship between stress and MHS was stronger among participants who were asymptomatic than among symptomatic participants. A study focused on the relationship between HIV symptoms and HRQoL found that individuals reporting symptoms were more likely to engage in spiritual activities such as prayer [58]. The fact that spiritual activity is associated with better adjustment to HIV-related stress has been demonstrated by previous research [59]. Perhaps, the negative relationship between stress and mental health was attenuated by better psychological adjustment among symptomatic participants. Accordingly, further research is needed to confirm this result and to explore further potential explanations.

We did not observe a significant difference in HRQoL between participants who received ART during follow-up and those who did not. Similar results with no differences in HRQoL between ART-treated and ART-naïve patients had been reported in previous studies [34, 60]. The effect of ART on HRQoL has been regarded as a balance between alleviation of symptoms and side effects [61]. Encountering side effects from an ART regimen in an early stage of treatment may contribute to the insignificant change in PHS. Additionally, this phenomenon might be explained by the fact that patients with more advanced HIV progression and worse immunological status were more likely to initiate ART. Also, it is possible that the median duration of ART is only 6 (IQR 4–9) months for our participants, which might be too short to find significant differences in HRQoL between two groups. A longer observation of the relationship between ART and HRQoL is needed.

For socio-demographic factors, we found that older age was associated with worse physical health. A similar finding has been certified by other studies [33, 56]. This finding is most likely due to older age being correlated with a decline in physical functioning of the body over time. We also found that participants with a rural household registration had lower PHS scores than participants who had an urban household registration, which might be because participants with a rural household registration were more economically unstable and had less access to medical care. It should be noted that GEE analysis results showed that participants who contacted HIV through IDU, blood products, or uncertain routes presented lower PHS, which may be caused by the relatively small sample size of this subgroup. Therefore, the HRQoL among these populations should be discussed in further studies.

There are several limitations to our study. First, because this study was based on consecutive samples in only one region, the generalizability of our findings is limited. Second, possibly due to work-related stigma, a higher rate of employment was found in participants who dropped out the follow-up survey, which might have an impact on the HRQoL estimates. Third, we were unable to measure all variables associated with HRQoL. Future studies should consider additional factors, such as coping styles and stigma. Another limitation is that our study’s follow-up of only 1 year might be rather short for people with HIV, a chronic disease. Further research with regular and longer periods of observation is needed to discover the impact of ART, adherence to ART, and other factors on HRQoL over a longer term.

## Conclusion

In conclusion, a relatively lower HRQoL among HIV-infected people shortly after HIV diagnosis and an increase in HRQoL were observed among people 1 year after HIV diagnosis. This finding indicates that additional attention should be paid to the HRQoL among HIV-infected people after HIV diagnosis. Physical and psychosocial factors such as HIV-related symptoms, depression, higher stress levels, and a lack of social support had a negative influence on HRQoL. These findings suggest the need to treat depression, reduce stress, and enhance social support at an early stage of HIV disease. Timely diagnosis and management of HIV-related symptoms are also crucial to optimize HRQoL among people with HIV infection. Additionally, special attention should be paid to those with a rural household registration and those of older age.
